# A New Epoxy-Based Layered Silicate Nanocomposite Using a Hyperbranched Polymer: Study of the Curing Reaction and Nanostructure Development

**DOI:** 10.3390/ma7031830

**Published:** 2014-03-04

**Authors:** Pilar Cortés, Iria Fraga, Yolanda Calventus, Frida Román, John M. Hutchinson, Francesc Ferrando

**Affiliations:** 1 Departament d’Enginyeria Química, ETSEIAT, Universitat Politècnica de Catalunya, Terrassa 08222, Barcelona, Spain; E-Mail: m.pilar.cortes@upc.edu; 2 Escola Universitària Salesiana de Sarrià, Passeig Sant Joan Bosco 74, 08017, Barcelona Spain; E-Mail: ifraga@euss.es; 3 Centre for NanoEngineering and Departament de Màquines i Motors Tèrmics, ETSEIAT, Universitat Politècnica de Catalunya, Terrassa 08222, Barcelona, Spain; E-Mails: calventus@mmt.upc.edu (Y.C.); roman@mmt.upc.edu (F.R.); 4 Department of Mechanical Engineering, Universitat Rovira i Virgili, C/Països Catalans 26, 43007, Tarragona, Spain; E-Mail: f.ferrando@urv.cat

**Keywords:** hyperbranched polymer (HBP), differential scanning calorimetry (DSC), exfoliation, epoxy, montmorillonite, impact energy, nanocomposites, layered silicate

## Abstract

Polymer layered silicate (PLS) nanocomposites have been prepared with diglycidyl ether of bisphenol-A (DGEBA) epoxy resin as the matrix and organically modified montmorillonite (MMT) as the clay nanofiller. Resin-clay mixtures with different clay contents (zero, two, five and 10 wt%) were cured, both isothermally andnon-isothermally, using a poly(ethyleneimine) hyperbranched polymer (HBP), the cure kinetics being monitored by differential scanning calorimetry (DSC). The nanostructure of the cured nanocomposites was characterized by small angle X-ray scattering (SAXS) and transmission electron microscopy (TEM), and their mechanical properties were determined by dynamic mechanical analysis (DMA) and impact testing. The results are compared with an earlier study of the structure and properties of the same DGEBA-MMT system cured with a polyoxypropylene diamine, Jeffamine. There are very few examples of the use of HBP as a curing agent in epoxy PLS nanocomposites; here, it is found to enhance significantly the degree of exfoliation of these nanocomposites compared with those cured with Jeffamine, with a corresponding enhancement in the impact energy for nanocomposites with the low clay content of 2 wt%. These changes are attributed to the different cure kinetics with the HBP, in which the intra-gallery homopolymerization reaction is accelerated, such that it occurs before the bulk cross-linking reaction.

## 1. Introduction

Polymer layered silicate (PLS) nanocomposites based on epoxy resins have been widely studied, and there are a number of excellent reviews of their preparation and properties [1–4], but the anticipated dramatic enhancement of the properties of the epoxy resin by the incorporation of small quantities of clay has not yet been realized. In large part, this has been due to the difficulty of achieving the desired nanostructure, in which the clay layers are exfoliated and distributed homogeneously throughout the epoxy matrix. In most cases, the nanostructure consists of both intercalated and exfoliated nanostructures, and the nanocomposite under these circumstances is usually referred to as partially exfoliated (e.g., [5–9]). For one of the most widely used epoxy resins, diglycidyl ether of bisphenol-A (DGEBA), this has proven to be a considerable drawback, as the degree of exfoliation is usually rather low [10–12].

One approach to overcoming this problem that has recently been investigated is to incorporate hyperbranched polymers (HBP) into these systems. HBPs have attracted considerable attention as modifiers of epoxy resins, principally for the purposes of toughening [13–19], though their effects on the morphology [20–22] and on other properties, such as the cure kinetics [23–25] and the thermal expansion coefficient and microhardness, have also been investigated [26]. The study of HBPs in epoxy composites in general [27] and in nanocomposites in particular has been more limited, most studies being on polyurethane nanocomposites [28–35], although there have been some studies on HBPs in epoxy-based nanocomposites systems [36–38], in which the epoxy is usually DGEBA.

The supposed advantages of incorporating an HBP in the nanocomposite system are both to improve the dispersion of the clay in the epoxy resin matrix and also to increase the degree of exfoliation that takes place when the system is cured. In this respect, we review briefly the results reported in [36–38]. In the work of Becker *et al*. [36], in which an epoxy-functionalized HBP was used in DGEBA-based nanocomposites, there still remain many clay tactoids, clearly visible by transmission electron microscopy (TEM), with a clay layer separation of approximately 10 nm. The system studied by Das *et al*. [37], in which a hyperbranched polyurea modified nanoclay was used in vegetable oil-modified sulfone epoxy nanocomposites, is so different from ours that we do not make further comment. Eissa *et al*. [38] fabricated DGEBA-based nanocomposites using an amino-terminated poly(ester-amine) HBP as a co-curing agent with a commercial amine hardener. For clay contents greater than 3 wt%, there are clear peaks in the small angle X-ray scattering (SAXS) scattering pattern, corresponding to about 3-nm *d*-spacing; for lower clay contents, the absence of peaks is not convincing, due to the substantial scatter, presumably as a consequence of insufficient exposure of the sample to the X-rays, while the TEM micrographs show clay agglomerations for the sample with5 wt% loading and are at too low a magnification to identify individual clay layers. These very few examples are therefore rather unconvincing with respect to the supposed advantages of incorporating HBP into the epoxy PLS nanocomposite system in order to improve the exfoliation of the nanostructure. Accordingly, in the present work, we attempt to show both how and why this use of HBP can indeed be advantageous, by fabricating PLS nanocomposites based upon the bi-functional epoxy resin, DGEBA, and a −NH_2_ terminated poly(ethyleneimine) hyperbranched polymer, which acts as the curing agent. The effect of the clay content is studied, and the results are compared also with previous work in which epoxy/clay nanocomposites were fabricated using the same DGEBA resin, but for which the curing agent was a polyoxypropylene diamine, Jeffamine D-230 [10].

## 2. Experimental

### 2.1. Materials

The epoxy resin used was a commercial bifunctional epoxy resin, diglycidyl ether of bisphenol-A (DGEBA), DER 331 (Dow Chemical Company), with an epoxy equivalent in the range 182–192 g/eq and a viscosity in the range 11,000–14,000 mPa·s at 25 °C.

The curing agent used was a commercial hyperbranched polymer (HBP), an –NH_2_-terminated poly(ethyleneimine), Lupasol PR8515 (BASF Española S.L., Tarragona, Spain), which has a viscosity at 20 °C in the range 10,000 to 20,000 mPa·s and an average molecular weight of 2000 g/mol.

The nanoclay used was a commercial organically modified clay, montmorillonite (MMT), Nanomer I.30E (Nanocor Inc., Arlington Heights, IL, USA), with a cation exchange capacity of 92 meq/100 g and in which the organic modifier is octadecylammonium.

**Figure 1.**
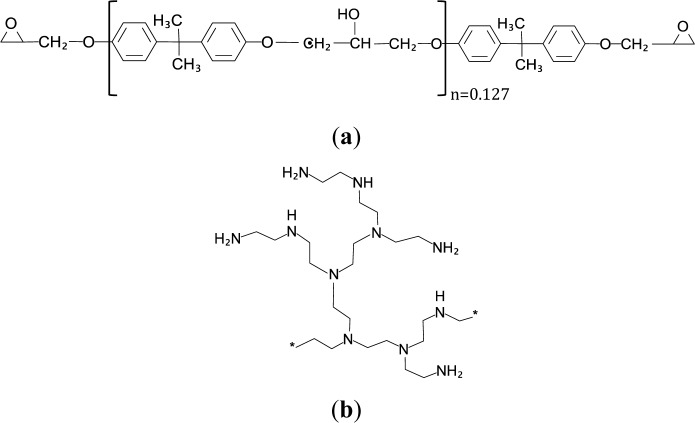
Chemical structures of (**a**) diglycidyl ether of bisphenol-A (DGEBA) and (**b**) a hyperbranched poly(ethyleneimine).

Figure 1 shows (a) the structure of the epoxy resin and (b) the idealized structure of a hyperbranched poly(ethyleneimine).

### 2.2. Preparation of the Nanocomposites

To prepare the nanocomposites, firstly, pre-mixes of resin and clay were prepared, usually with small clay contents of 2 wt%, 5 wt% and 10 wt%. The preparation was performed in 3 stages: initially, the nanoclay and the resin were placed in a vessel and mixed on a hotplate using a magnetic stirrer (Jenway 1103, Bibby Scientific Limited, Stone, Staffordshire, UK) at a temperature in the range 40 to 50 °C for a period of 30 minutes, followed by mixing in an ultrasonic bath (Branson 3510, Emerson Industrial Automation, Soest, Netherlands) for 3 h at 45 °C, in order to improve the dispersion of the clay in the resin. Finally, the samples were drastically dispersed using a sonicator (Branson S450, Emerson Industrial Automation, Danbury, CO, USA) in pulse mode and at 30% amplitude for a total of 9.0 min, with a program of 6 separate steps of 1.5 min each, in which the sample was sonicated in three pulses of 30 s duration, with 30 s between them. The maximum temperature was limited to 45 °C by immersing the container, in which the sonication was taking place, in an ice/salt bath and allowing up to 1 h between the 1.5 min steps for the temperature to reduce sufficiently.

Once the resin/clay pre-mixes had been prepared, the curing agent was added, in a stoichiometric ratio. For the differential scanning calorimetry (DSC) samples, the curing agent was mixed in by hand rapidly in small quantities on a watch glass, at room temperature. For the bulk samples, these mixtures were immediately degassed under vacuum at room temperature, ready for the curing reaction at 110 °C for a period of 1 h in an oven.

### 2.3. Optical Microscopy

The dispersion of the clay in the resin was observed using a Leica polarizing transmission optical microscope (Leica Microsystems GmbH, Wetzlar, Germany). A small quantity of each mixture was placed on a glass slide with a cover slip and viewed between crossed polars at 10× magnification in the objective lens plus 10× magnification in the eyepiece.

### 2.4. Thermal Analysis

The cure kinetics of the prepared samples was monitored by DSC in non-isothermal scans at 2, 5, 10, 15 and 20 °C/min and isothermal scans at the temperatures of 50 °C, 70 °C, 80 °C and 90 °C. The equipment used was a Mettler-Toledo DSC 821e differential scanning calorimeter (Mettler Toledo AG, Analytical, Schwerzenbach, Switzerland) equipped with a sample robot and Haake EK90/MT intracooler (Thermo Electron GmbH, Karlsruhe, Germany). Additional isothermal scans at the temperature of 50 °C were made using a stochastic temperature modulated DSC technique, TOPEM (Mettler-Toledo DSC 823e, Schwerzenbach, Switzerland), in order to determine the vitrificationtime [39,40]. All DSC curing experiments were performed with a dry nitrogen gas flow of 50 mL/min, and the DSC was calibrated for both heat flow and temperature using indium. The data evaluation was performed with the STAR^e^ software (Mettler Toledo AG, Schwerzenbach, Switzerland). For all the experiments, a small sample, of about 8–10 mg for standard DSC and about 20 mg for TOPEM, was weighed into an aluminum pan, sealed and immediately inserted into the DSC furnace, which was previously heated either to the start temperature for non-isothermal scans or to the curing temperature for isothermal experiments, whereupon the curing experiment was immediately started.

### 2.5. Nanostructural Characterization

The nanostructure of the cured nanocomposites was examined by small angle X-ray scattering (SAXS) and by transmission electron microscopy (TEM). For SAXS, cured bulk samples were converted to powder in a ball mill (Retsch model MM 400, Retsch GmbH, Haan, Germany) using 20-mm diameter steel balls and a frequency of 20 Hz for a period of 4 minutes. A Bruker D8 Advanced diffractometer (Bruker Corporation, Billerica, MA, USA) was used to obtain the scattering diagram, measurements being taken in a range of 2θ = 1° to 8° with copper Kα radiation, the scans being made with steps in 2θ of 0.02° and with a time of 10 s for each step.

TEM was carried out with a Jeol Jem-2010 High Resolution electron microscope (Jeol Ltd., Tokyo, Japan), with an accelerating voltage of 200 kV. Samples were prepared by ultramicrotomy of the bulk cured nanocomposites, to give a section of about 50-nm thickness.

### 2.6. Dynamic Mechanical Analysis

For the dynamic mechanical analysis, the degassed mixture of resin/clay plus curing agent (HBP) was poured into a cylindrical mold, approximately 10 mm in diameter and 11 mm-long, and was cured in an air-circulating oven at 110 °C for 1 h. Samples in the form of thin discs, approximately 6 mm in diameter and 1.5 mm-thick, were then machined from the molded cylinders. The storage modulus (*G*′) and the mechanical loss factor (tan δ = *G*″/*G*′) were determined as a function of temperature and frequency by dynamic mechanical analysis (DMA) using the Mettler-Toledo model DMA861e (Mettler Toledo AG, Analytical, Schwerzenbach, Switzerland) in shear mode. DMA measurements were made over a range of frequencies (0.1, 0.5, 1, 5, 10, 40, 100, 500 and 1000 Hz) and at a 2 °C/min heating rate over the temperature range from 25 °C to 180 °C.

### 2.7. Impact Testing

For the impact tests, the degassed mixture of resin/clay plus curing agent (HBP) was poured into a mold, consisting of nine separate rectangular cavities of the required dimensions for subsequent machining, so as to provide multiple samples for the impact tests, and was cured in an air-circulating oven at 110 °C for 1 h. The impact tests were performed at room temperature using a ZwickIzod impact tester 5110 according to a standard test method [41] on rectangular specimens, machinedto 25 × 12 × 2.5 mm from the bulk molded samples. The impact tester had a hammer with an energy of 0.545 J, and a minimum of five specimens were tested for each composition.

## 3. Results

### 3.1. Dispersion (Optical Microscopy)

The polarizing optical microscopy shows a somewhat inhomogeneous dispersion of the clay in the resin, with a large number of agglomerations of the order of 30–40 μm in size, with the largest being of the order of 60 μm. For the three compositions studied, the dispersion is slightly better for the2 wt% clay content. The existence of these agglomerations, which has been noted previously for the same epoxy resin and clay mixtures [42], is one of the reasons why it is difficult to achieve a high degree of exfoliation in this nanocomposite system; if there are significant agglomerations, then the resin cannot penetrate into all the clay galleries, or the curing agent is inhibited from reacting with the epoxy resin, or the clay layers are physically prevented from separating. There are ways in which this dispersion can be improved. One is to use the so-called slurry preparation method [43–48], in which the clay is first dispersed in a solvent before adding the resin and ultimately removing the solvent under vacuum, and another is to pre-condition the resin-clay mixture [10,11,42,49,50]. Here, we do not adopt these procedures, as the objective is to compare the use of the HBP as the curing agent with the same nanocomposite system using the polyoxypropylene diamine curing agent, Jeffamine D-230 [42], for which the same clay agglomerations were present.

### 3.2. Thermal Analysis (DSC)

**Figure 2.**
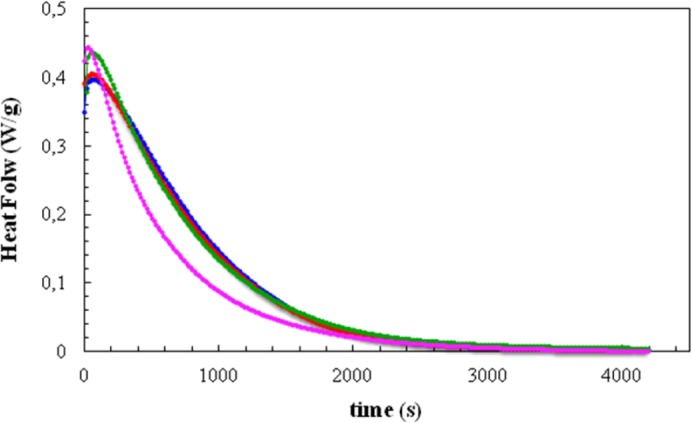
DSC scans for the isothermal cure at 70 °C of the resin/hyperbranched polymer (HBP) without clay (blue curve) and for the nanocomposites with 2 wt% (red), 5 wt% (green) and 10 wt% (pink) clay content.

Figure 2 shows the DSC curves, in the form of heat flow per gram of total mass of the sample, including clay, plotted as a function of time for isothermal cure at 70 °C. Four isothermal cure curves are shown here: for the resin/HBP system alone and for the same system with the three different clay contents.

It can be seen that the addition of the clay generally causes an advance in the reaction. For the clay contents of 2 wt% and 5 wt%, this is evident from the increase in the peak heat flow and a small shift to shorter times of the right-hand flank of the cure curve, though the time at which the peak heat flow occurs remains constant at 56 s for these clay contents. On the other hand, for the 10 wt% clay content there is a much more marked shift of the cure curve to shorter times, with the peak heat flow also occurring at the much shorter time of 28 s. It is quite clear, therefore, that the addition of the modified clay has a significant catalytic effect on the cure reaction; this effect was previously observed in the same resin and clay system, but cured with Jeffamine D-230 (Huntsman Corp., Salt Lake City, UT, USA) [10].

Second and third (non-isothermal) scans were made following cure at each of the isothermal cure temperatures, *T*_c_, for all the samples, in order to determine the residual heat of reaction and the glass transition temperature of the fully cured system, respectively. The results are gathered in Table 1 with respect to the glass transition temperature, *T*_g∞_, of the final nanocomposite after the third scan, as well as the total heat of reaction (Δ*H*) per epoxy equivalent (ee), calculated as the sum of the partial and residual heats.

**Figure 3.**
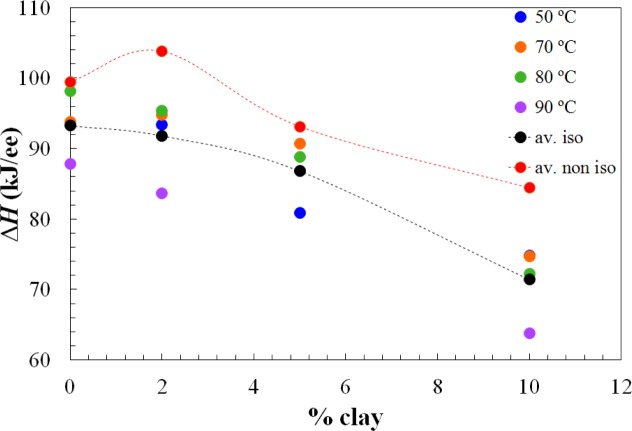
The dependence of the total heat of reaction, Δ*H*, on clay content, for the isothermal cure of nanocomposites cured with Lupasol HBP. Dotted black and red lines represent the average values for each percent of clay content, for isothermal (av. iso) and non-isothermal cure (av. non iso), respectively.

Apart from the effect of the clay content on the advance in the cure reaction, increasing the clay content also results in a decrease in the total heat of the reaction, as can be seen from the results in the table, presented graphically in Figure 3. This effect of clay content has been observed previously also for the same nanocomposite systems cured with Jeffamine D-230 [10]. There is inevitably a certain amount of scatter in these results as a consequence of some heat of the reaction that can be lost at the start of the isothermal experiment, because of some reaction that takes place during the time between the addition of the curing agent and the initiation of the isothermal cure. This is particularly relevant in the nanocomposites prepared with the Lupasol HBP, as the reaction begins very quickly for this system. In this respect, it is worth pointing out that each value given in Table 1 corresponds to a separate experiment in which the required amount of Lupasol is added to the epoxy-clay pre-mix. There is not a masterbatch of epoxy-clay-Lupasol from which samples are taken for curing isothermally at different temperatures; indeed, for the 2 wt% samples, there was even more than one epoxy-clay pre-mix used to obtain these results. The scatter in the results presented in the table can be associated with this preparation procedure, which is essential on account of the high reactivity of the Lupasol, but notwithstanding this it is possible to observe the trends mentioned above clearly.

**Figure 4.**
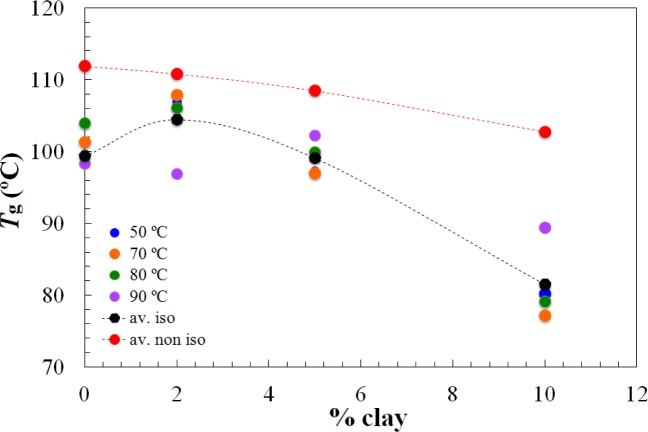
The dependence of the glass transition temperature of the fully cured samples, *T*_g∞_, on clay content, for the isothermal cure of nanocomposites with Lupasol HBP at the isothermal temperatures indicated. The black dotted line represents the average value for each percent of clay content; the red dotted line represents the average values for the non-isothermal cure experiments on the same nanocomposites.

On the other hand, the *T*_g∞_ of the fully cured nanocomposites is highest for the 2 wt% clay content and decreases significantly for higher clay contents, as shown in Figure 4. It is interesting to note that this is significantly different from the situation for the nanocomposites cured with Jeffamine D-230, for which there was a monotonic decrease in the glass transition temperature with increasing clay content, both for the partially cured samples after the first isothermal cure and for the *T*_g∞_ of the fully cured nanocomposites after the second (non-isothermal) scan. This is indicative of a significant nanostructural difference between the nanocomposites prepared using the different curing agents, particularly at the clay content of 2 wt%. Further evidence for this nanostructural difference will be presented in the non-isothermal cure results.

The values of the vitrification time during the isothermal cure (at 50 °C) for the different clay contents have been determined by TOPEM; vitrification occurs when the glass transition of the curing system reaches the isothermal cure temperature. The results are given in Table 2, which shows that the vitrification advances as the clay content increases. This is because the reaction is advanced by the addition of clay, so that the glass transition of the curing system increases more rapidly for the higher clay contents.

Results are given here in Table 2 only for 50 °C, the lowest of the four isothermal cure temperatures used in this work. The reason for selecting the lowest cure temperature for this study of the vitrification time is a practical one and related to the way in which the vitrification time is determined by TOPEM. This procedure requires the characterization, in the trace of the quasi-static specific heat capacity, *c*_p0_, of the liquid-like and glassy states before and after vitrification, respectively, such that the asymptotic behavior in each of these regions can be defined [39,40]. For higher cure temperatures, the vitrification advances significantly, such that it is not possible to define unequivocally theliquid-like asymptotic region, and hence the vitrification time cannot be determined here with sufficient accuracy for isothermal cure temperatures higher than 50 °C. This difficulty is particularly noticeable for the present system with HBP, which is highly reactive.

**Figure 5.**
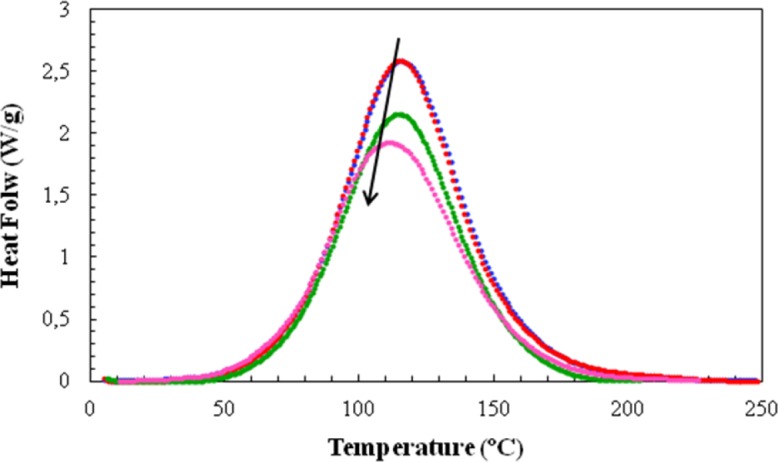
DSC scans for the non-isothermal cure at 20 °C/min of the resin/HBP system without clay (blue curve) and for the nanocomposites with 2 wt% (red), 5 wt% (green) and 10 wt% (pink) clay content. The arrow indicates the direction of increasing clay content.

For the non-isothermal cure experiments, Figure 5 shows a typical set of curves of specific heat flow, referred to the total mass of the sample, including the mass of the clay, as a function of temperature for four of the systems studied, at a heating rate of 20 °C/min. Similar to the case for the isothermal experiments in Figure 2, the addition of clay results in an advance of the reaction, such that the peak heat flow occurs at lower temperatures as the clay content increases. The heat of the reaction and the temperature for maximum heat flow (*T*_p_) are found from such curves, while the second scan gives the final glass transition temperature of the non-isothermally cured nanocomposite (*T*_g∞_) and verifies that the heat of the reaction is indeed the total heat, in that there is no significant residual curing reaction. The data obtained in this way for the non-isothermal cure of all the nanocomposite systems and for all heating rates, *q*, are summarized in Table 3.

**Figure 6.**
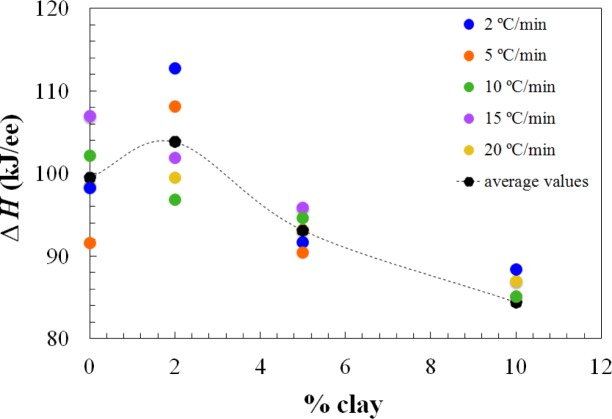
The dependence of the total heat of the reaction, Δ*H*, on clay content for the non-isothermal cure. The dotted line represents the average value for each percent of clay content.

**Figure 7.**
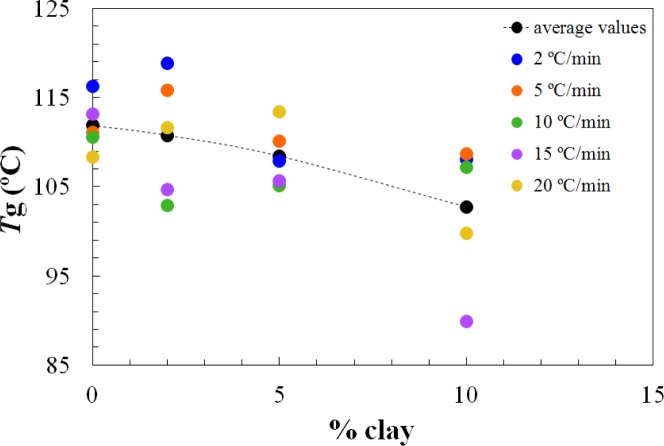
The dependence of the glass transition temperature of the fully cured samples, *T*_g∞_, on clay content for the non-isothermal cure. The black dotted line represents the average value for each percent of clay content.

The dependences of the heat of the reaction and *T*_g∞_ on the clay content for non-isothermal cure are shown in Figures 6 and 7, respectively. The average values for all the heating rates are also compared with the average values for isothermal cure in Figures 3 and 4. It can be seen that both the heat of the reaction and *T*_g∞_ tend to decrease with increasing clay content, a trend that was observed earlier for the same nanocomposite system cured with Jeffamine [10]. This behavior is also similar to the trend displayed for isothermal cure in Figures 3 and 4, though for non-isothermal cure it is the heat of the reaction that appears to pass through a maximum at 2 wt% clay, whereas for isothermal cure, it is *T*_g∞_ that shows a maximum at this clay content. Taken together, these results indicate that the thermal properties of the 2 wt% clay content nanocomposite are improved relative to the unreinforced system and also relative to the systems with higher clay contents, which is attributed to the better dispersion of the clay in the resin by sonication.

The most important aspect of the non-isothermal cure curves shown in Figure 5, though, is their symmetry. In previous work with the same DGEBA epoxy and clay system, but cured withJeffamine [10], these non-isothermal cure curves always displayed a pronounced shoulder on the high temperature flank of the exotherm. This was attributed to a homopolymerization reaction taking place within the clay galleries. This intra-gallery reaction is, in principle, beneficial for the exfoliation of the clay, but for cure with Jeffamine this reaction is occurring after the majority of the extra-gallerycross-linking reaction. The exfoliation of the clay layers is therefore inhibited by the surrounding rigid cured matrix. This was confirmed by the SAXS results and by TEM, where significant layer stacking is observed, with *d*-spacings even less than that of the intercalated clay as a consequence of the contraction that takes place during crosslinking. On the contrary, the present results shown in Figure 5 do not display any such shoulder, implying either that the intra-gallery homopolymerization reaction has been accelerated by the presence of the HBP, not being visible in these curves because it is now hidden beneath the overall exotherm due to the cross-linking, or that it no longer occurs, the reaction taking place within the clay galleries now being a cross-linking reaction. This interpretation is supported both by the SAXS results, discussed immediately below, which do not show any scattering peak for the epoxy/clay/HBP system, and also by the TEM results, for which it will be shown that much better exfoliation is achieved when Lupasol HBP is used as the curing agent in this nanocomposite system.

### 3.3. Small Angle X-Ray Scattering (SAXS)

**Figure 8.**
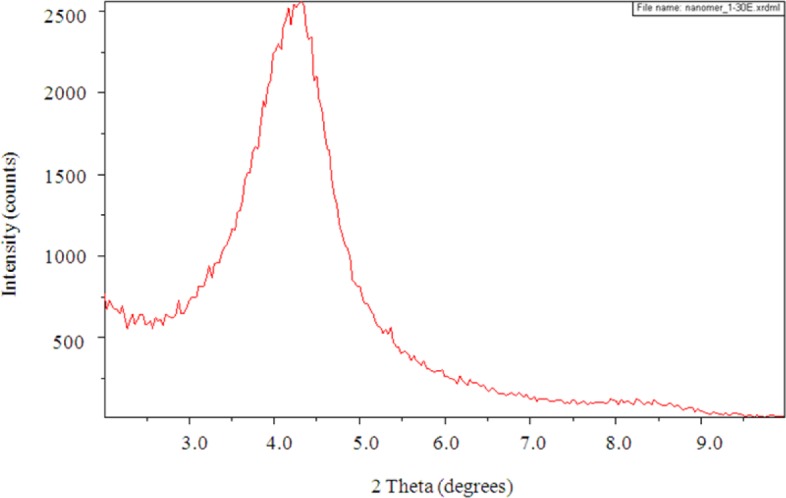
The small angle X-ray scattering (SAXS) diffraction pattern for the organically modified clay, montmorillonite I.30E.

**Figure 9.**
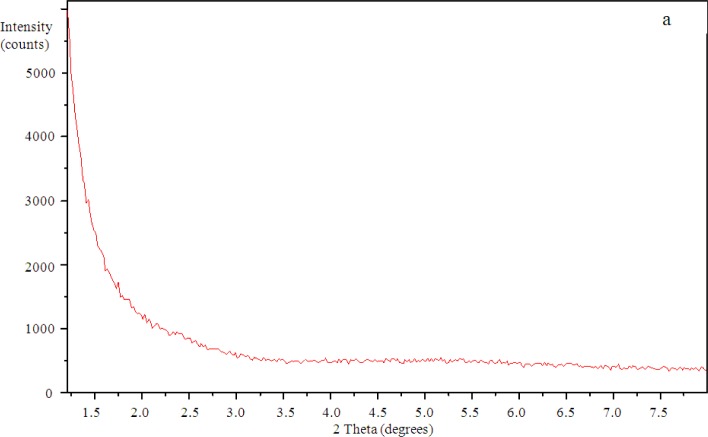

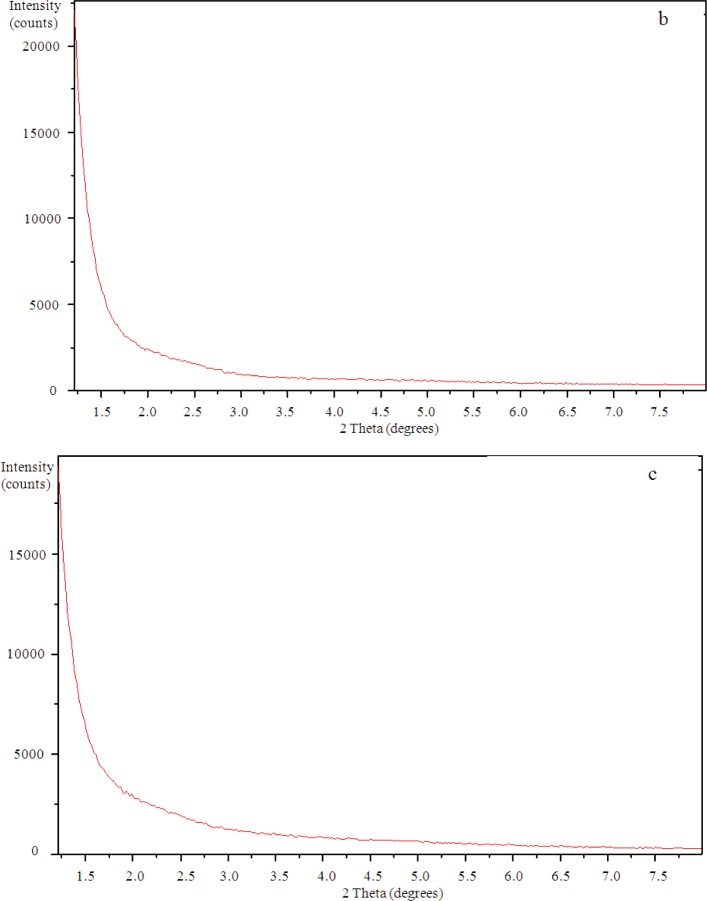
The SAXS diffraction pattern for the DGEBA-Lupasol nanocomposite with three different clay contents: (**a**) 2 wt%; (**b**) 5 wt%; (**c**) 10 wt%.

The SAXS results show a peak in the scattering intensity for the organically modified clay at a scattering angle of 2θ just less than 4.4°, corresponding to a *d*-spacing of 2.1 nm, as can be seen in Figure 8. This *d*-spacing increases to between 3.6 and 3.7 nm for the resin/clay mixture withoutHBP [42], indicating that the epoxy resin has intercalated between the clay layers. After the addition of the HBP and effecting the cure schedule, the scattering pattern for this cured resin/clay/HBP system shows that this peak has disappeared, as can be seen in Figure 9a for the nanocomposite containing 2 wt% clay, suggesting that the nanocomposite cured with HBP is better exfoliated than that cured with Jeffamine and, indeed, that it is exfoliated to the extent that the *d*-spacing has increased beyond the limit of detection by this technique (about 8 nm). Similar scattering patterns are found also for the nanocomposites containing the higher clay contents of 5 wt% and 10 wt%, and these are also included in Figure 9. This is a major improvement on the results obtained for the DGEBA/clay/Jeffamine system, for which a significant scattering peak always remained in the SAXS diffractogram [10], sometimes even occurring at a *d*-spacing of about 1.4 nm, significantly less than that of the intercalated clay and less even than that of the original organically modified clay, which can be attributed to the effect of the contraction on the cure of the epoxy resin matrix surrounding the clay agglomerates.

### 3.4. Transmission Electron Microscopy (TEM)

**Figure 10.**
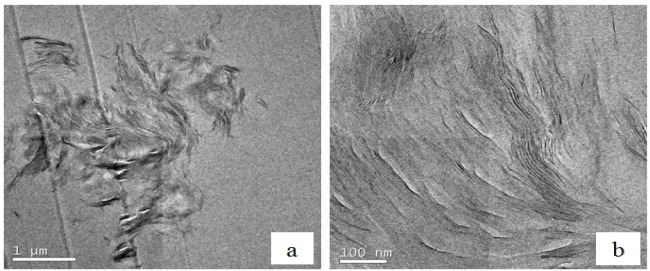
TEM micrographs at low magnification of DGEBA nanocomposite with 2 wt% clay and cured with Lupasol HBP: (**a**) one of the agglomerations in the sample; (**b**) higher magnification of the interior of the agglomeration in (**a**).

Further confirmation of this partially exfoliated nanostructure in the nanocomposites fabricated with the HBP can be seen in the TEM micrographs of Figure 10 for cured nanocomposites containing 2 wt% clay. Figure 10a, at low magnification, shows one of the clay agglomerations remaining after the cure, the size of this particle being about 4 μm. This is clearly much less than the average agglomerate size of 30 to 40 μm that was observed by optical microscopy to exist in the uncured resin/clay mixture, implying that the cure process with HBP not only leads to the separation of the clay layers within the agglomerates, as will be seen shortly, but also breaks down the agglomerates themselves. From these low magnification TEM micrographs, it is possible to ascertain that the HBP is more effective than is Jeffamine in reducing the agglomerate size in this way. Within this same agglomeration, at the higher magnification shown in Figure 10b, it can be seen that there are some regions in which the clay layers are still in a certain register and other regions in which either the layer separation is much greater or the register between clay layers has been lost altogether.

**Figure 11.**
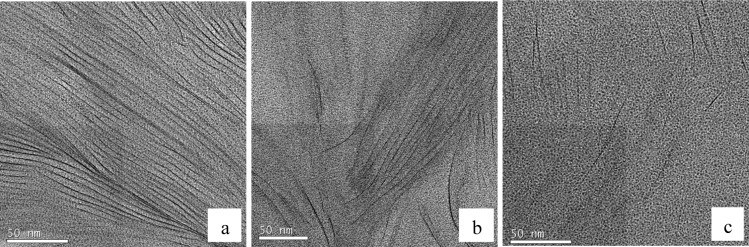
TEM micrographs at high magnification of three different zones within the agglomerate of Figure 10a for a DGEBA nanocomposite with 2 wt% clay and cured with Lupasol HBP.

At even higher magnification, it is possible to identify the individual clay layers. This is shown in Figure 11a–c, for three different regions within the same agglomeration viewed in Figure 10. The region shown in Figure 11a shows that there still remains some layer stacking in these cured nanocomposites, with little evidence of any exfoliation in this particular region, the *d*-spacing being approximately within the range of 3.6 to 3.7 nm, the same spacing as that determined by SAXS for the intercalated clay. On the other hand, the region shown in Figure 11b shows a significant proportion of clay layers that are more widely separated, the *d*-spacing lying in the range of six to 7 nm or more in this figure, and many layers are rather randomly dispersed; in other words, with a significant degree of exfoliation. This is seen to an even greater extent in Figure 11c, where only a few clay layers are visible within the same area, now with almost no register at all between them, and with separations of up to 20 to 50 nm or more.

### 3.5. Dynamic Mechanical Analysis (DMA)

**Figure 12.**
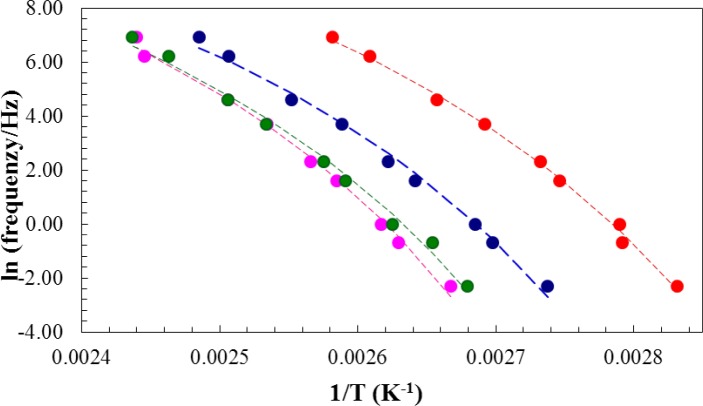
Ln(frequency/Hz) *versus* reciprocal glass transition temperature derived from the maximum in tan δ in scans at 2 °C/min for nanocomposite samples with different clay contents: 0 wt%, blue; 2 wt%, pink; 5 wt%, green; 10 wt%, red. The dashed lines represent the fit of the Vogel–Tammann–Fulcher (VTF) equation.

From the measurements of tan δ as a function of temperature during the DMA scans at 2 °C/min, the glass transition temperature, determined mechanically, was identified as the temperature at which the maximum in tan δ occurred. The glass transition temperature was observed to increase with increasing frequency, and the plot of ln(frequency) *versus* reciprocal glass transition temperature is shown in Figure 12. Here, it can be seen that the mechanical glass transition temperature increases for both the 2 wt% and 5 wt% nanocomposites with respect to the system with no clay and then decreases significantly for the nanocomposite with 10 wt% clay. This behavior is similar to that found by DSC, and shown in Table 1 and Figure 4, though the increase in the glass transition temperature determined by DMA is more marked for the 2 wt% nanocomposite and also extends to the clay content of 5 wt%. This supports our earlier conclusion that the thermal properties of these nanocomposites are enhanced at low clay contents.

It is possible to obtain a good fit to the data in Figure 12 with a Vogel–Tammann–Fulcher (VTF) equation of the form:

$$\ln(f)=A-\frac{B}{T-T_{0}}$$ (1)

in which *f* is the frequency, *A* and *B* are constants and *T*_0_ is the Kauzmann temperature [51,52]. The best fit curves to each set of data for the different clay contents are shown by the dashed lines in Figure 12. From this fitting procedure, the values of the parameters, *A*, *B* and *T*_0_, are obtained and are given in Table 4. The most significant aspect of these results is that the temperature, *T*_0_, is between 40 °C and 50 °C below the calorimetric glass transition temperature for the isothermally cured samples (see Table 1 and Figure 4), as is commonly found, and that it increases for the 2 wt% and 5 wt% nanocomposites, in agreement with the increase in the glass transition temperature. The other parameters, *A* and *B*, do not show any significant or systematic dependence on the clay content.

### 3.6. Impact Testing

The average results of the impact tests are shown in Table 5, together with the 95% confidence interval for each. It should be noted that the unusually large uncertainty for the 5 wt% samples is a consequence of testing only five different specimens for this clay content, in contrast to eight or nine different specimens tested for the other clay contents. What can be seen from these results is that there is a significant increase in the impact energy for the 2 wt% clay content. This is consistent with the thermal analysis results, which showed that the thermal properties of the 2 wt% sample were improved with respect to both the unreinforced sample and the samples with higher clay contents; and also with the nanostructural characterization, where it was seen that the 2 wt% sample showed a significant degree of exfoliation, necessary for the enhancement of the fracture properties of the nanocomposite. For higher clay contents, the impact energy decreases again, to approximately the same value as that for the unreinforced system. This is probably a consequence of the competition between the reinforcement resulting from the exfoliated clay layers and the opposite effect resulting from the clay agglomerations, which continue to have a significant presence with respect to the impact strength at these higher clay contents.

## 4. Conclusions

Polymer layered silicate (PLS) nanocomposites based upon DGEBA epoxy resin and organically modified montmorillonite clay have been fabricated using a hyperbranched polymer (HBP) as the curing agent. In comparison with earlier work in which the same system was cured with a diamine, the effect of the HBP is to modify the cure kinetics and, in particular, to accelerate the intra-gallery homopolymerization reaction, which permits a greater degree of exfoliation to occur. The SAXS results show no scattering peaks, and in the TEM micrographs the clay agglomerations can be seen to be smaller in size and better dispersed, with a nanostructure in which the clay layers are well separated and with little layer stacking, particularly for a low clay loading of 2 wt%, but also to some extent for clay loadings up to 10 wt%. These nanostructural improvements at the 2 wt% clay loading correlate with an increase in the glass transition temperature, determined both calorimetrically and by dynamic mechanical analysis and, in particular, with an increase in the impact energy.

## Figures and Tables

**Table 1.** DSC results for the isothermal cure of nanocomposites with HBP: *T*_c_ is the cure temperature; *T*_g∞_ is the glass transition temperature of the fully cured nanocomposite and ∆*H* is the total heat of the reaction.

*T*_c_ (°C)	Clay content							
	0 wt%		2 wt%		5 wt%		10 wt%	

	*T*_g∞_ (°C)	Δ*H* (kJ/ee)	*T*_g∞_ (°C)	Δ*H* (kJ/ee)	*T*_g∞_ (°C)	Δ*H* (kJ/ee)	*T*_g∞_ (°C)	Δ*H* (kJ/ee)
50	–	–	106.9	93.4	97.3	80.9	80.3	74.9
70	101.3	93.8	107.9	94.8	97.0	90.7	77.2	74.7
80	104.0	98.1	106.1	95.4	99.9	88.8	79.1	72.3
90	98.3	87.8	96.8	83.7	102.3	86.8	89.4	63.8

**Table 2.** Vitrification time for the different clay contents during isothermal cure at 50 °C.

**Clay Content**	0 wt%	2 wt%	5 wt%	10 wt%
**Vitrification time (min)**	76.5	76.4	71.8	35.1

**Table 3.** DSC results for non-isothermal cure of nanocomposites cured with HBP at the indicated heating rates, *q: T*_p_ is the temperature for maximum heat flow; *T*_g∞_ is the glass transition temperature of the fully cured sample and Δ*H* is the total heat of the reaction.

*q* (°C/min)	Clay content											
	0 wt%			2 wt%			5 wt%			10 wt%		

	*T*_p_ (°C)	*T*_g∞_ (°C)	Δ*H* (kJ/ee)	*T*_p_ (°C)	*T*_g∞_ (°C)	Δ*H* (kJ/ee)	*T*_p_ (°C)	*T*_g∞_ (°C)	Δ*H* (kJ/ee)	*T*_p_ (°C)	*T*_g∞_ (°C)	Δ*H* (kJ/ee)
2	76.2	116.3	98.2	77.0	118.8	112.7	75.8	107.9	91.7	74.6	108.9	88.4
5	89.3	111.1	91.6	91.7	115.8	108.1	90.3	110.1	90.4	88.3	108.7	85.1
10	104.3	110.6	102.6	101.7	102.9	96.8	102.6	105.1	94.6	99.3	107.2	85.1
15	111.7	113.1	106.9	110.4	104.7	101.9	109.7	105.7	95.8	104.9	89.9	76.6
20	116.4	108.4	98.5	115.9	111.6	99.5	115.3	113.4	93.2	112.1	98.7	87.0

**Table 4.** Dependence of the VTF parameters on the clay content in nanocomposites cured with Lupasol.

Clay (wt%)	A	B (K)	*T*_0_ (K)
**0**	18.5	1062	322.1
**2**	19.2	1093	332.1
**5**	18.4	1044	330.4
**10**	19.9	1115	309.5

**Table 5.** Impact energy results for the nanocomposites cured with Lupasol HBP.

Clay Content	0 wt%	2 wt%	5 wt%	10 wt%
**Impact energy (kJ/mm^2^**)	1.61 ± 0.04	2.08 ± 0.06	1.57 ± 0.80	1.69 ± 0.15

## References

[1] Alexandre M., Dubois P. (2000). Polymer-layered silicate nanocomposites: Preparation, properties and uses of a new class of materials. Mater. Sci. Eng.

[2] Ray S.S., Okamoto M. (2003). Polymer/layered silicate nanocomposites: A review from preparation to processing. Progr. Polym. Sci.

[3] Becker O., Simon G.P. (2005). Epoxy layered silicate nanocomposites. Adv. Polym. Sci.

[4] Karak N. (2006). Polymer (epoxy) clay nanocomposites. J. Polym. Mater.

[5] Brown J.M., Curliss D., Vaia R.A. (2000). Thermoset-layered silicate nanocomposites: Quaternary ammonium montmorillonite with primary diamine cured epoxies. Chem. Mater.

[6] Becker O., Cheng Y-B., Varley R.J., Simon G.P. (2003). Layered silicate nanocomposites based on various high-functionality epoxy resins: The influence of cure temperature on morphology, mechanical properties, and free volume. Macromolecules.

[7] Chen C.G., Curliss D. (2003). Preparation, characterization, and nanostructural evolution of epoxy nanocomposites. J. Appl. Polym. Sci.

[8] Velmurugan R., Mohan T.P. (2004). Room temperature processing of epoxy-clay nanocomposites. J. Mater. Sci.

[9] Mohan T.P., Kumar M.R., Velmurugan R. (2005). Rheology and curing characteristics of epoxy-clay nanocomposites. Polym. Int.

[10] Montserrat S., Román F., Hutchinson J.M., Campos L. (2008). Analysis of the cure of epoxy based layered silicate nanocomposites: Reaction kinetics and nanostructure development. J. Appl. Polym. Sci.

[11] Pustkova P., Hutchinson J.M., Román F., Montserrat S. (2009). Homopolymerization effects in polymer layered silicate nanocomposites based upon epoxy resin: Implications for exfoliation. J. Appl. Polym. Sci.

[12] Lipinska M., Hutchinson J.M. (2012). Elastomeric epoxy nanocomposites: Nanostructure and properties. Comp. Sci. Technol.

[13] Boogh L., Pettersson B., Månson J.-A.E. (1999). Dendritic hyperbranched polymers as tougheners for epoxy resins. Polymer.

[14] Wu H., Xu J., Liu Y., Heiden P. (1999). Investigation of readily processable thermoplastic-toughened thermosets. V. Epoxy resin toughened with hyperbranched polyester. J. Appl. Polym. Sci.

[15] Mezzenga R., Månson J.-A.E. (2001). Thermo-mechanical properties of hyperbranched polymer modified epoxies. J. Mater. Sci.

[16] Xu G., Shi W., Gong M., Yu F., Feng J. (2004). Curing behavior and toughening performance of epoxy resins containing hyperbranched polyester. Poly. Adv. Tech.

[17] Jin F.-L., Park S.-J. (2006). Thermal properties and toughness performance of hyperbranched-polyimide-modified epoxy resins. J. Polym. Sci. B Polym. Phys.

[18] Flores M., Fernandez-Francos X., Ferrando F., Ramis X., Serra A. (2012). Efficient impact resistance improvement of epoxy/anhydride thermosets by adding hyperbranched polyesters partially modified with undecenoyl chains. Polymer.

[19] Luo L.J., Meng Y., Qiu T., Li X.Y. (2013). An epoxy-ended hyperbranched polymer as a new modifier for toughening and reinforcing in epoxy resin. J. Appl. Polym. Sci.

[20] Mezzenga R., Boogh L., Pettersson B., Månson J.-A.E. (2000). Chemically induced phase separated morphologies in epoxy resin-hyperbranched polymer blends. Macromol. Symp.

[21] Mezzenga R., Plummer C.J.G., Boogh L., Månson J.-A.E. (2001). Morphology build-up in dendritic hyperbranched polymer modified epoxy resins: Modeling and characterization. Polymer.

[22] Ratna D., Simon G.P. (2001). Thermomechanical properties and morphology of blends of a hydroxyl-functionalized hyperbranched polymer and epoxy resin. Polymer.

[23] Ratna D., Simon G.P. (2001). Thermal and mechanical properties of a hydroxyl-functional dendritic hyperbranched polymer and trifunctional epoxy resin blends. Polym. Eng. Sci.

[24] Xu G., Shi W., Shen S. (2004). Curing kinetics of epoxy resins with hyperbranched polyesters as toughening agents. J. Polym. Sci. B Polym. Phys.

[25] Frigione M., Calò E. (2008). Influence of an hyperbranched aliphatic polyester on the cure kinetic of a trifunctional epoxy resin. J. Appl. Polym. Sci.

[26] Morell M., Ramis X., Ferrando F., Yu Y., Serra A. (2009). New improved thermosets obtained from DGEBA and a hyperbranched poly(ester-amide). Polymer.

[27] Mezzenga R., Boogh L., Månson J.-A.E. (2001). A review of dendritic hyperbranched polymer as modifiers in epoxy composites. Comp. Sci. Technol.

[28] Plummer C.J.G., Garamszegi L., Leterrier Y., Rodlert M., Månson J.-A.E. (2002). Hyperbranched polymer layered silicate nanocomposites. Chem. Mater.

[29] Rodlert M., Plummer C.J.G., Grünbauer H.J.M., Månson J.-A.E. (2004). Hyperbranched polymer/clay nanocomposites. Adv. Eng. Mater.

[30] Rodlert M., Plummer C.J.G., Garamszegi L., Leterrier Y., Grünbauer H.J.M., Månson J.-A.E. (2004). Hyperbranched polymer/montmorillonite clay nanocomposites. Polymer.

[31] Rodlert M., Plummer C.J.G., Leterrier Y., Månson J.-A.E., Grünbauer H.J.M. (2004). Rheological behavior of hyperbranched polymer/montmorillonite clay nanocomposites. J. Rheol.

[32] Maji P.K., Guchhait P.K., Bhowmick A.K. (2008). Effect of the microstructure of a hyperbranched polymer and nanoclay loading on the morphology and properties of novel polyurethane nanocomposites. Appl. Mater. Interfaces.

[33] Deka H., Karak N. (2009). Vegetable oil-based hyperbranched thermosetting polyurethane/clay nanocomposites. Nano. Res. Lett.

[34] Deka H., Karak N. (2010). Influence of highly branched poly(amido amine) on the properties of hyperbranched polyurethane/clay nanocomposites. Mater. Chem. Phys.

[35] Deka H., Karak N. (2011). Bio-based hyperbranched polyurethane/clay nanocomposites: Adhesive, mechanical, and thermal properties. Polym. Adv. Tech.

[36] Ratna D., Becker O., Krishnamurthy R., Simon G.P., Varley R.J. (2003). Nanocomposites based on a combination of epoxy resin, hyperbranched epoxy and a layered silicate. Polymer.

[37] Das G., Deka H., Karak N. (2013). Bio-based sulfonated epoxy/hyperbranched polyurea-modified MMT nanocomposites. Int. J. Polym. Mater. Polym. Biomater.

[38] Eissa M.M., Youssef M.S.A., Ramadan A.M., Amin A. (2013). Poly(ester-amine) hyperbranched polymer as toughening and co-curing agent for epoxy/clay nanocomposites. Polym. Eng. Sci.

[39] Fraga I., Montserrat S., Hutchinson J.M. (2008). Vitrification during the isothermal cure of thermosets. Part 1. An investigation using TOPEM, a new temperature modulated technique. J. Therm. Anal. Calorim.

[40] Fraga I., Montserrat S., Hutchinson J.M. (2008). Vitrification during the isothermal cure of thermosets: Comparison of theoretical simulations with temperature-modulated DSC and dielectric analysis. Macromol. Chem. Phys.

[41] (2008). ASTM D4508-05 Standard Test Method for Chip Impact Strength of Plastics.

[42] Hutchinson J.M., Montserrat S., Román F., Cortés P., Campos L. (2006). Intercalation of epoxy resin in organically modified montmorillonite. J. Appl. Polym. Sci.

[43] Lu J., Ke Y., Qi Z., Yi X. (2001). Study on intercalation and exfoliation behavior of organoclays in epoxy resin. J. Polym. Sci. B Polym. Phys.

[44] Ton-That M.-T., Ngo T.-D., Ding P., Fang G., Cole K.C., Hoa S.V. (2004). Epoxy nanocomposites: Analysis and kinetics of cure. Polym. Eng. Sci.

[45] Chen B., Liu J., Chen H.B., Wu J.S. (2004). Synthesis of disordered and highly exfoliated epoxy/clay nanocomposites using organoclay with catalytic function via acetone-clay slurry method. Chem. Mater.

[46] Miyagawa H., Rich M.J., Drzal L.T. (2004). Amine-cured epoxy/clay nanocomposites. I. Processing and chemical characterization. J. Polym. Sci. B Polym. Phys.

[47] Wang K., Chen L., Wu J.S., Toh M.L., He C.B., Yee A.F. (2005). Epoxy nanocomposites with highly exfoliated clay: Mechanical properties and fracture mechanisms. Macromolecules.

[48] Liu W., Hoa S.V., Pugh M. (2005). Organoclay-modified high performance epoxy nanocomposites. Comp. Sci. Tech.

[49] Benson Tolle T., Anderson D.P. (2004). The role of preconditioning on morphology development in layered silicate thermoset nanocomposites. J. Appl. Polym. Sci.

[50] Hutchinson J.M., Shiravand F., Calventus Y. (2012). Intra- and extra-gallery reactions in tri-functional epoxy polymer layered silicate nanocomposites. J. Appl. Polym. Sci.

[51] Hodge I.M. (1994). Enthalpy relaxation and recovery in amorphous materials. J. Non Cryst. Sol.

[52] Hutchinson J.M. (1995). Physical aging of polymers. Progr. Polym. Sci.

